# Relationship Between Sleep Duration and Psychosocial Well-Being in Healthcare Personnel: Identification of Predictors and Vulnerability Patterns

**DOI:** 10.3390/bs15091290

**Published:** 2025-09-22

**Authors:** Eva Urbón, Carlos Salavera, José M. López-Chamorro, Almudena F. Diaz-Carrasco

**Affiliations:** 1 Department of Psychology and Sociology, University of Zaragoza, 50009 Zaragoza, Spain; eurbon@unizar.es (E.U.); salavera@unizar.es (C.S.); 2 Cátedra TEA Ediciones, University of Zaragoza, 50009 Zaragoza, Spain; 3 Health Research Centre and Department of Education, University of Almería, 04120 Almería, Spain; 4 Department of Psychology, University of Almería, 04120 Almería, Spain; 5 Faculty of Education and Social Work, University of Valladolid, 47002 Valladolid, Spain

**Keywords:** sleep, burnout, dysfunctional coping, allergies, eating behaviours, predictors, vulnerability patterns, healthcare personnel

## Abstract

The present study examined the relationship between sleep duration and eating behaviours, stress symptoms, and burnout in healthcare professionals. Objective: The present study aimed to examine whether sleep duration influenced the psychosocial well-being of healthcare personnel, as well as to identify possible predictors and patterns of vulnerability in this population. Method: A cross-sectional study was conducted with a sample of 194 public healthcare workers (mainly women and nursing staff). Validated questionnaires were used: the EAT-40, the EDI, the MBI, and a stress symptom scale. The participants were classified into two groups according to their sleep duration (fewer than six hours of sleep and six hours or more of sleep). Results: A sleep duration of fewer than six hours was associated with higher levels of depersonalisation (burnout), physical and emotional symptoms of stress (fatigue, tachycardia, memory loss, crying easily), dysfunctional coping strategies (self-medication, isolation), and more restrictive eating behaviours. A regression analysis identified seven predictors of sleep duration: allergies, marital status, hours worked, depersonalisation, alcohol consumption, interpersonal distrust, and skipping meals, which together explained 18% of the variance. A network analysis showed positive correlations between these variables in the group with a shorter sleep duration, indicating a pattern of cumulative psychosocial vulnerability. Conclusions: Although the cross-sectional design limits causal inference, the results underscore the importance of sleep as a key factor in the emotional and functional well-being of healthcare personnel. Organisational interventions focused on promoting rest, emotional management, and stress prevention are suggested, considering sleep not only as a biological need, but also as a relevant indicator of psychosocial health for healthcare quality.

## 1. Introduction

Human daily activity is regulated by the biological clock, which synchronises circadian rhythms with both internal and environmental factors. This synchronisation is mediated by the suprachiasmatic nucleus, a brain structure that responds mainly to stimuli such as light, sleep, and food (De Martino, 2013; Khan et al., 2018; Rovira-Llopis et al., 2024).

Among these factors, sleep is fundamental. It is a basic biological need, characterised by a decrease in physical activity, alterations in consciousness, and physiological changes. It is essential for restoring the balance of the nervous system, enabling protein synthesis, and repairing the body, and it also contributes to psychological well-being (Datta & MacLean, 2007; Bae et al., 2019; Urbón, 2020).

The health of healthcare workers can be affected by altered sleep patterns, which are often disrupted by shift work, especially night shifts, as well as 24 h shifts. Various studies have explained how healthcare workers who work shifts have numerous health problems (Cuartero et al., 2005; Gander et al., 2019; Gifkins et al., 2020; Creasy et al., 2022; Easton et al., 2024). Sleep problems in these workers are related to other issues that they suffer from: fatigue, emotional instability, anxiety, depression, eating disorders, and certain risk behaviours, such as smoking, alcohol consumption, or the use of stimulants (Loria et al., 2009; van de Langenberg et al., 2019; Sachdeva & Goldstein, 2020; Wolska et al., 2022).

Not getting enough sleep or having poor-quality sleep is also associated with metabolic problems, eating problems (maladaptive behaviours and/or dysfunctional eating), and emotional and relational problems (Caruso, 2014; Yeo & Koh, 2022).

Sleeping between five and seven hours per night has been associated with an increase in body mass index, appetite dysregulation, a higher likelihood of obesity, an increased mortality risk, metabolic diseases, and emotional health problems (Ko et al., 2007; Spiegel et al., 2009; Cappuccio et al., 2010; Itani et al., 2017; Franco et al., 2012; Givens et al., 2015; Liu et al., 2016; Yin et al., 2017; Chaput et al., 2018; Cheng & Zhang, 2020; Liu & Chen, 2020). The time of intake also influences metabolic and circadian regulation. Several studies have shown that people who eat lunch before 3 p.m. lose more weight than those who eat later, highlighting the importance of the timing of food intake (Arble et al., 2009; Jakubowicz, 2013). At the hormonal level, a sleep restriction reduces leptin levels and increases ghrelin, which stimulates appetite and contributes to weight gain (Spiegel et al., 2004; Taheri et al., 2004; Penev, 2007; van Egmond et al., 2023; McHill et al., 2023). However, a recent meta-analysis found no significant changes in these hormone levels after acute sleep deprivation (Gresser et al., 2025).

Finally, the relationship between sleep, stress, and burnout must also be considered in this worker profile. Stress is another key factor in the effects of sleep deprivation. Recent studies have highlighted that stress raises cortisol levels, which compromises emotional regulation and exacerbates the negative effects of a circadian misalignment in healthcare personnel (Weiss & Mullaney, 2020; Lombardo & Leone, 2021; Rogers & Bailey, 2021; Lemieux & Rowland, 2022). Sleeping for fewer than 7 h makes workers between 8.33 and 17.18 times more likely to experience burnout (Saintila et al., 2024), which indicates a relationship between hours of sleep and burnout.

Given this scenario, and in order to clarify the discrepancies presented in relation to studies on sleep duration and its relationship with psychosocial well-being, the following hypotheses were proposed:

**H1.**  *those who sleep for fewer hours will have a reduced psychosocial well-being, with behaviours more compatible with eating disorders and stress*.

**H2.**  *personal, work, eating pattern, and stress variables may act as predictors and patterns of vulnerability in the sleep hours of healthcare personnel*.

The independent variable (IV) to be considered was sleep hours (Group 1 = fewer than six hours and Group 2 = six or more hours of sleep) and the dependent variables (DVs) analysed were eating behaviours, stress symptoms, and burnout. The overall objective was to examine whether the duration of sleep in healthcare personnel influences their psychosocial well-being, specifically in the areas of eating behaviours, stress symptoms, and burnout, as well as to identify possible predictors and patterns of vulnerability.

Three specific objectives were determined: (O1) to examine the association between sleep duration and the areas of psychosocial well-being identified: eating behaviours (inefficiency, skipping meals), stress symptoms (fatigue, cognitive difficulties, emotional responses), and levels of burnout (especially depersonalisation); (O2) to identify significant predictors of sleep duration, considering personal, work-related, behavioural, and psycho-emotional variables; and (O3) to model interconnected patterns of psychosocial vulnerability using network analysis techniques to visualise how somatic, emotional, and behavioural factors reinforce each other.

## 2. Methodology

### 2.1. Study Design

First, the management team of the hospitals was contacted to request permission and administer the questionnaires to healthcare personnel. Once the project was deemed feasible by the hospitals’ research committee, fieldwork began.

After obtaining the necessary permissions, communication was established with hospital unit managers to begin administering the questionnaires. The participants were informed in writing of the objectives through an information letter that they had to complete with their details, expressing their consent to participate and the privacy of their responses. The letter also informed them about the rules for completing the questionnaire and how, when, and where they should submit it. Each centre had an accessible place where workers could submit the questionnaires anonymously. The data collection period was one month.

### 2.2. Participants

The population of this study belonged to the city of Zaragoza (Spain) and the main sector of work was public health. The range of health professionals included in this study comprised medical and nursing staff working at four hospitals in the city.

The inclusion criteria used were as follows: working at local public health centres in Zaragoza (Spain), working as medical or nursing staff, and completing all questionnaires correctly. A total of 837 healthcare professionals were invited to participate in the research. The initial number of participants was 213, of whom 19 were excluded for not completing the questionnaire battery properly or not meeting the inclusion criteria. Finally, the sample consisted of 194 participants.

### 2.3. Measurements

**Sociodemographic and dietary intake and eating habits questionnaire.** An ad hoc questionnaire was designed to assess different characteristics of the participants with regard to the following variables: age, sex, marital status, number of children, height, weight, position, unit, age, length of service, hours worked per week, time spent working shifts, meals consumed, skipped meals, and foods eaten.

The questionnaire consisted of 76 questions, and although there were open-ended and closed-ended questions, most of them were multiple-choice questions. In our study, the Cronbach’s alpha coefficient was 0.81.

**Eating Attitudes Test** (Garner & Garfinkel, 1979; Spanish adaptation by Castro et al., 1991).

This is an instrument developed to detect eating disorders in the general population, as well as to identify current or incipient cases of anorexia and bulimia nervosa. It assesses abnormal eating attitudes related to a fear of gaining weight, the urge to lose weight, or the presence of restrictive eating patterns. It contains 40 items, grouped into seven factors: bulimic behaviours, body image with a tendency towards thinness, the use or abuse of laxatives, the presence of vomiting, food restrictions, secret eating, and a perceived social pressure to gain weight. Based on these factors, three main subscales were extracted: diet (related to avoiding fattening foods and concern about being thinner), bulimia and concern about food (about bulimic behaviours and thoughts about food), and oral control (self-control imposed on eating behaviour and the perception that others exert pressure on the person to gain weight). These factors showed an internal consistency (Cronbach’s alpha) of 0.90, 0.83, and 0.84, respectively.

This is a self-administered 40-item test (EAT-40) that is scored on a 6-point Likert scale ranging from never to always. The score range is 0 to 120 points, with a cut-off point of 30 points or higher. It is an easy-to-use instrument with a high reliability, sensitivity, and cross-cultural validity.

The Spanish adaptation of the EAT-40, used in this study, was validated in a group of patients with anorexia nervosa and a healthy control group, showing an internal consistency of 0.93 for the total and 0.92 for the group of patients with anorexia. In our sample, Cronbach’s alpha was 0.77, showing an acceptable internal consistency. For the factors of diet, bulimia, and concern about food and oral control, the internal consistency was 0.42, 0.66, and 0.60, respectively.

**Eating disorder inventory** (Garner et al., 1983; Spanish adaptation by Corral et al., 1998).

This is a self-administered instrument designed to assess different cognitive and behavioural areas of anorexia and bulimia nervosa. In the present study, it was used to assess different behaviours and attitudes towards food, weight, and body image.

It consists of 64 items grouped into eight subscales: motivation to lose weight, bulimic symptoms, body dissatisfaction, ineffectiveness and low self-esteem, perfectionism, interpersonal distrust, interoceptive awareness, and fear of growing up. The first three subscales measure behaviours and attitudes towards food, weight, and body image (motivation to lose weight, bulimic symptoms, dissatisfaction with one’s body image); the imbalances expressed in these areas are not specific to anorexia nervosa, as similar responses appear in groups of people concerned about their diet. The other five subscales (ineffectiveness and low self-esteem, perfectionism, interpersonal distrust, interoceptive awareness or identification, and fear of growing up) assess general psychological characteristics associated with eating disorders. Each item is scored on a 6-point Likert scale. All subscales can be added together for an overall score, or each subscale can be used separately. Clinically, the quantitative value of each of the eight subscales is more relevant than the overall score. The total maximum score on the questionnaire is 192, with a cut-off point of 42 or more points on the eight original subscales to diagnose an eating disorder (Garner et al., 1983).

In the Spanish version, the internal consistency was between 0.83 and 0.92, except for the fear of maturing subscale, the internal consistency of which was 0.65.

In our sample, Cronbach’s alpha for the motivation to lose weight subscale was 0.67; for the bulimia subscale, it was 0.69; for the body dissatisfaction subscale, it was 0.71; for the ineffectiveness subscale, it was 0.69; for the perfectionism subscale, it was 0.72; for interpersonal distrust, it was 0.71; for interoceptive awareness, it was 0.62; and for the fear of growing up, it was 0.70.

**Burnout inventory** (Maslach Burnout Inventory, Maslach & Jackson, 1986).

This questionnaire assesses the degree of professional burnout syndrome. It consists of 22 items in the form of statements about the feelings, thoughts, and attitudes of professionals in their work and towards their patients/clients, which are answered according to the frequency with which the professional experiences them, choosing from a scale ranging from 0 (“never”) to 6 (“daily”). The items are grouped into three subscales that assess different aspects of burnout: emotional exhaustion, depersonalisation or cynicism, and low personal achievement or ineffectiveness. The burnout score is obtained by adding the points obtained in the emotional exhaustion and depersonalisation subscales; finally, the score obtained in the personal achievement subscale is subtracted from this sum.

The emotional exhaustion subscale refers to the experience of feeling emotionally drained by work demands. It is measured using nine items. The maximum score is 54 points. The internal consistency in the Spanish adaptation was 0.87. In our research, the resulting consistency was 0.85.

The depersonalisation subscale assesses the degree to which individuals recognise attitudes of coldness and detachment. It consists of five items and the maximum score is 30 points. The internal consistency of this subscale in the Spanish adaptation was 0.85, while in our sample, the consistency was 0.77.

The achievement subscale, consisting of eight items, assesses feelings of self-efficacy and personal fulfilment at work. The maximum score is 48 points. The consistency of the subscale in the Spanish adaptation (Salanova et al., 2000) was 0.78, while in the present study, it was 0.74.

**Inventory of stress-related symptoms** (Payne & Firth-Cozens, 1987).

This assesses a series of physical/behavioural symptoms that may be related to work stress through 36 items.

The subjects had to indicate the symptoms they had experienced during the last year using a dichotomous scale (yes/no), obtaining a total score for the number of stress-related symptoms. The symptoms to be analysed were memory loss, difficulty maintaining attention, forgetfulness or absent-mindedness, negative self-talk, boredom, mental confusion, disorganised thoughts, excessive worry, difficulty concentrating, lack of new ideas, hyperactivity, fatigue, allergies, headaches, tachycardia or arrhythmias, irregular breathing, skin problems, gastrointestinal disorders, eating disorders, sexual dysfunction, sleep problems, depression, isolation, irritability, anxiety, panic attacks, mood swings, impatience, crying easily, overreacting, feeling sensitive, getting discouraged easily, back pain, overeating, alcohol and tobacco abuse, substance use, and self-medication. In our research sample, a Cronbach’s alpha of 0.89 was obtained.

### 2.4. Data Analysis

For the analysis of the assessment instruments, we used the SPSS 29 software package for Windows (IBM, 2022).

In all the statistical tests, the bilateral significance level was set at *p* < 0.05. The Kolmogorov–Smirnov test was also used to test the normality of the data set. As some variables did not follow a normal distribution, it was necessary to adjust the scores obtained for these variables using logarithms.

For the statistical analysis, the sample was divided into two groups (fewer than six hours of sleep and six hours or more of sleep), considering this as an independent variable. Student’s *t*-test was used for continuous variables (e.g., clinical scales of eating and burnout) and the χ^2^ test was used for categorical variables (e.g., stress symptoms). In addition, odds ratios (ORs) and their corresponding *p*-values were obtained. Finally, a linear regression analysis was performed to determine which variables could be most related to the workers’ hours of sleep. Finally, the JASP software, v. 0.10.2 (JASP Team, 2023), and the Fruchterman–Reingold algorithm (Fruchterman & Reingold, 1991) were used to perform undirected and weighted network analyses.

## 3. Results

The sample analysed consisted of 194 participants, of whom 85.05% were women and 14.95% were men. Most had been working for more than five years (61.9%) and the majority were married (59.8%).

In terms of job position, almost half were nurses (47.96%), followed by nursing assistants (32.97%) and doctors (19.07%).

With regard to body mass index (BMI), the overall average was 23.99 kg/m^2^ (SD = 3.78), which is within the normal weight range. It was observed that, within the group that had fewer than 6 h of sleep, the average BMI was slightly higher (24.08) compared to the group that slept more than 6 h (23.55). In terms of nutritional status, the overweight group had an average BMI of 28.41 kg/m^2^, with a predominance of women (63.5%) compared to men (36.5%).

The average number of children was 1.11 (SD = 1.06), and was higher in the group that slept for fewer than 6 h (1.20) compared to those who slept 6 or more hours (0.65), a difference that appears to be statistically significant (***). A higher proportion of single people was also observed in the group that slept more than 6 h (58.1%) compared to the other group (31.9%).

Overall, the data show a predominantly female sample, with a high representation of nursing staff, and a general trend towards normal weight, although with a not-insignificant proportion of overweight individuals. Some variables, such as the number of children and the marital status, appear to be associated with the number of hours of sleep (Table 1).

**Table 1:** Characterisation of the sample.

*Variables*	*Fewer Than 6 h* *(n = 163)*	*More Than 6 h* *(n = 31)*	*Total* *(N = 194)*
BMI (Kg/m^2^) *	24.08 (3.85)	23.55 (3.45)	23.99 (3.78)
Normal weight	21.88 (1.65)	21.84 (1.73)	21.87 (1.66)
Women	103 (95.37%)	22 (95.6%)	125 (95.4%)
Men	5 (4.63%)	1 (4.4%)	6 (4.6%)
Overweight	28.46 (2.10)	28.40 (3.25)	28.41 (3.09)
Women	33 (60%)	7 (87.5%)	40 (63.5%)
Men	22 (40%)	1 (12.5%)	23 (36.5%)
No. of children *	1.20 (1.06) ^(^*^)(^**^)^	0.65 (0.91)	1.11 (1.06)
Sex **			
Women	136 (88.61%)	29 (93.5%)	165 (85.05%)
Men	27 (11.39%)	2 (6.5%)	29 (14.95%)
Marital status **			
Single	52 (31.9%)	18 (58.1%)	70 (36.1%)
Married	103 (63.2%)	13 (41.9%)	116 (59.8%)
Separated	7 (4.3%)	-	7 (3.6%)
Widowed	1 (0.6%)	-	1 (0.5%)
**Job title**			
Doctor	31 (19.01%)	6 (19.35%)	37 (19.07%)
Nurse	80 (49.07%)	14 (45.16%)	94 (47.96%)
Auxiliary nurse	52 (31.92%)	11 (35.49%)	63 (32.97%)
Length of service **			
More than five years	103 (63.2%)	17 (54.8%)	120 (61.9%)
Fewer than five years	60 (36.8%)	14 (45.2%)	74 (38.1%)

Note: * Cells represent means and standard deviations; ** cells represent frequencies and percentages with respect to the same group; CI: 95% confidence interval; The results obtained on the clinical scales showed significant differences between the groups according to the number of hours of sleep (fewer than or more than six hours), with some statistically significant differences (Table 2).

**Table 2:** The *t*-test clinical scales according to hours of sleep.

Clinical Scales	*Fewer Than Six Hours* *(n = 163)*	*More Than Six Hours* *(n = 31)*	*t*	*p*	*Total* *(N = 194)*
Burnout (Depersonalisation)	6.13 (5.07) ^(^*^)(^*^)^	4.09 (3.74)	2.66	0.009	4.39 (4.04)
EAT-40 (Ineffectiveness)	1 (1.12)	1.69 (2.68) ^(^*^)(^**^)^	−1.40	0.001	1.58 (2.51)
EAT-40 (Interpersonal Mistrust)	1.52 (1.38)	2.07 (2.23) ^(^*^)(^*^)^	−1.32	0.01	1.98 (2.13)
EDI (Diet)	2.16 (3.18) *	1.38 (2.6)	1.47	0.04	1.5 (2.73)

Note: Cells represent means and standard deviations for each group; CI: 95% confidence interval; * *p* < 0.05; ** *p* < 0.01.

**Burnout—depersonalisation:** The group that slept for fewer than six hours had a significantly higher score for depersonalisation (M = 6.13; s.d. = 5.07) compared to those who slept more than six hours (M = 4.09; s.d. = 3.74), with this difference being statistically significant (t = 2.66, *p* = 0.009). This finding suggests a greater presence of symptoms of emotional exhaustion or a distant attitude towards patients in the group with less sleep.

**EAT-40—ineffectiveness:** Contrary to expectations, those who slept more than six hours reported higher levels of ineffectiveness (M = 1.69; s.d. = 2.68) compared to those who slept less (M = 1.00; s.d. = 1.12), with a significant difference (t = −1.40, *p* = 0.001). Although it may seem paradoxical, this could be related to other psychosocial or work variables that influence self-perceived competence, but in general, the group that slept more hours had more feelings of general incompetence, insecurity, emptiness, self-contempt, and lack of control over their own lives.

**EAT-4—interpersonal distrust:** People who slept more than six hours also scored higher for interpersonal distrust (M = 2.07; s.d. = 2.23) than those with fewer hours of sleep (M = 1.52; s.d. = 1.38). This difference was statistically significant (*p* = 0.01), which could suggest that rest does not necessarily imply less tension in interpersonal relationships within the workplace.

**EDI—diet:** In terms of restrictive eating behaviours, measured using the EDI diet subscale, a slightly higher tendency was observed in those who slept less (M = 2.16; s.d. = 3.18) compared to those who slept more (M = 1.38; s.d. = 2.60). This difference was also significant (*p* = 0.04), and it could be associated with higher levels of stress or dysregulation in healthy habits among those who sleep less.

Table 3 shows how the results reflect a higher prevalence of emotional, cognitive, and physical symptoms in the group that slept for fewer than six hours compared to those who slept more, with several of these differences being statistically significant (*p* < 0.05 or lower).

Cognitive and emotional symptoms: People who sleep for fewer than six hours reported more frequent memory loss (39.7% vs. 7.2%), difficulty concentrating (35.1% vs. 6.7%), and negative self-talk (23.2% vs. 4.1%), with significant differences. A higher prevalence of depressive symptoms such as crying easily (14.9% vs. 3.1%) and discouragement (20.6% vs. 3.6%) was also observed.

Physical symptoms: A higher frequency of fatigue (42.3% vs. 7.7%), tachycardia or arrhythmias (15.5% vs. 3.1%), and skin problems (22.7% vs. 4.1%) was recorded in the group with fewer hours of sleep. All these symptoms showed statistically significant associations, suggesting a physiological impact of a sleep deficit.

Stress-related behaviours: Isolation (9.3% vs. 1.5%) and self-medication (7.7% vs. 1.5%) were also more frequent among those who slept less, which could indicate maladaptive coping attempts.

Although the odds ratios (ORs) remained close to 1 with wide confidence intervals that included unity (indicating weak or inconclusive associations from a strictly statistical point of view), the differences in the proportions and *p*-values suggest a clear trend: a sleep deficit is associated with greater emotional distress, somatic symptoms, and dysfunctional coping strategies.

Table 4 shows the steps followed to incorporate the explanatory variables into the relationship between personal and work aspects, eating behaviours, and stress according to hours of sleep. The results of the regression analysis revealed a significant relationship between hours of sleep and allergies (F(1, 192) = 7.48, *p* = 0.007, with an R^2^ = 0.039); marital status (F(1, 191) = 6.62, *p* = 0.01, with an R^2^ = 0.072); hours worked per week (F(1, 190) = 4.79, *p* = 0.03, with an R^2^ = 0.094); depersonalisation (F(1, 189) = 4.94, *p* = 0.02, with an R^2^ = 0.117); alcohol consumption (F(1, 188) = 4.53, *p* = 0.03, with an R^2^ = 0.139); interpersonal distrust (F(1, 187) = 5.13, *p* = 0.02, with an R^2^ = 0.161); and skipping meals (F(1, 186) = 4.16, *p* = 0.04, with an R^2^ = 0.180). In particular, the findings indicated that these variables explain 18% of the variance in the number of hours of sleep.

In summary, the results highlight that allergies as a symptom of stress, marital status, hours worked per week, depersonalisation as a determining variable of *burnout*, alcohol consumption during the week, interpersonal distrust as a subscale of the EDI questionnaire, and skipping meals are the relevant predictors of the number of hours of sleep, with stress symptom allergies being the factor with the greatest impact.

**Figure 1:**
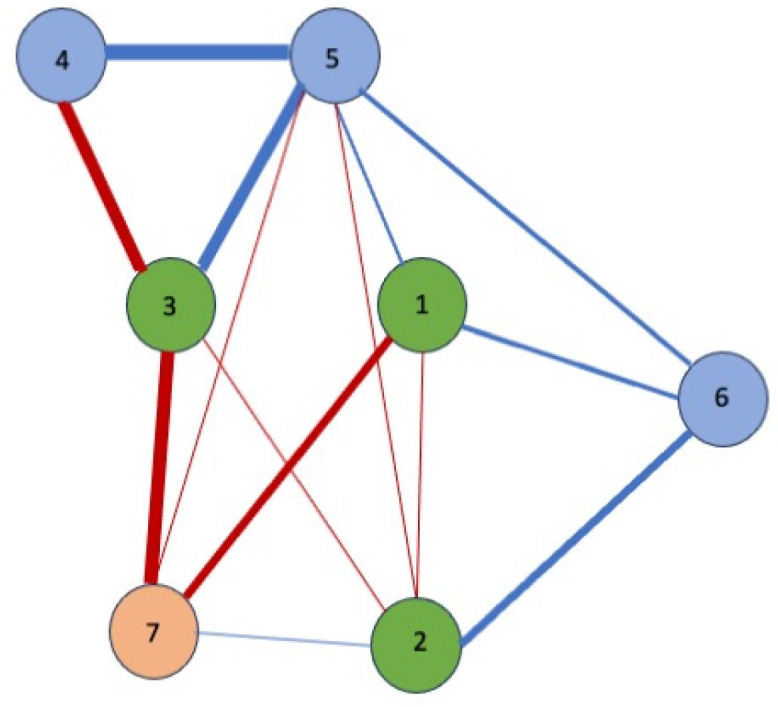
Workers who sleep more than six hours. **Personal aspects:** 1, alcohol consumption; 2, marital status; 3, sleep duration **Stress:** 4, interpersonal distrust; 5, depersonalisation; 6, allergies. **Eating behaviours:** 7, meal skipping.

**Figure 2:**
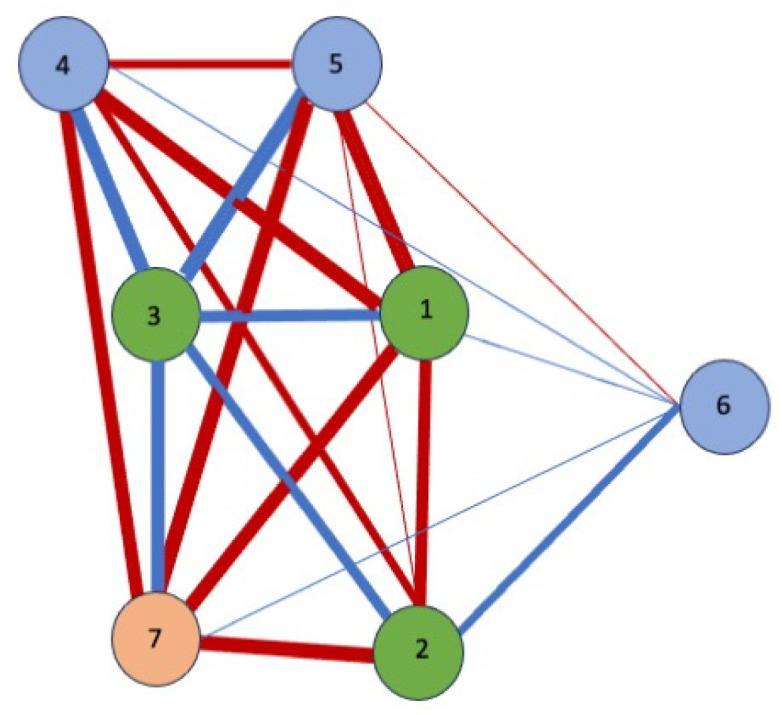
Workers who sleep for fewer than six hours. **Personal aspects:** 1, alcohol consumption; 2, marital status; 3, sleep duration **Stress:** 4, interpersonal distrust; 5, depersonalisation; 6, allergies. **Eating behaviours:** 7, meal skipping.

Finally, a network analysis (Figure 1 and Figure 2) was conducted to explore the relationships between the seven variables that best predict hours of sleep. The blue edges or lines represent positive correlations between the nodes, indicating that an increase in the score of one of the items leads to an increase in the score of the connected items. On the other hand, the red lines indicate negative correlations between nodes. Some variables showed unexpected correlations. In Figure 2, representing the correlations of variables in the group of workers who slept for fewer than 6 h, weekly alcohol consumption showed strong correlations with allergies as a symptom of stress, with meal skipping, with depersonalisation, with interpersonal distrust, and with marital status. Similarly, in Figure 1 (workers sleeping more than 6 h), depersonalisation was correlated positively with interpersonal distrust and alcohol consumption.

As for negative correlations, meal skipping was associated with marital status and allergies in workers who sleep for fewer hours (Figure 2), while the strongest negative correlations were between alcohol consumption and marital status and distrust (Figure 1).

These results suggest that less sleep leads to significant weekly alcohol consumption and is positively related to allergies as a symptom of stress (r = 0.80), depersonalisation (r = 0.78), skipping meals (r = 0.70), interpersonal distrust (r = 0.58), and not having a partner (r = 0.58).

## 4. Discussion

The results of this study provide relevant evidence on the association in a sample of healthcare professionals between the number of hours of sleep and psychosocial well-being in its various variables: personal, occupational, emotional, and behavioural.

Based on a detailed analysis of the data, it was observed that the duration of night-time rest not only affects physical health, but also has a significant impact on emotional well-being, perceptions of self-efficacy, coping styles, and dietary habits among healthcare workers. This result is consistent with previous research highlighting the fundamental role of sleep in emotional regulation, burnout, and overall mental health (Sørengaard et al., 2021; Saintila et al., 2024). In particular, professionals who slept for fewer hours reported more symptoms of emotional exhaustion and a distant attitude towards patients. In addition, they reported higher levels of emotional and cognitive distress, the presence of stress-related physical symptoms, and maladaptive behaviours. These findings support the findings of Urbón (2020) and Datta and MacLean (2007), who highlighted the restorative role of sleep in emotional and cognitive balance, as well as its relationship with increased depersonalisation and depressive symptoms when rest is insufficient. Along the same lines, the studies by Cuartero et al. (2005), Loria et al. (2009), and Bae et al. (2019) reinforce this perspective by pointing out that poor sleep can lead to anxiety, nervousness, depression, and difficulties in interpersonal relationships, which is consistent with the results found on discouragement, negative verbalisations, social isolation, and crying easily. Finally, recent research has directly linked work stress to emotional disturbances (Lemieux & Rowland, 2022; Rogers & Bailey, 2021; Salavera & Urbón, 2024; Weiss & Mullaney, 2020), a link that is particularly relevant in the hospital setting in which this study was conducted.

The sociodemographic characterisation of the sample allowed the findings to be contextualised. The group was predominantly female, with a high representation of nursing staff, most of whom had been working for more than five years. The average body mass index was within the normal weight range, although a subgroup with significant overweight was identified, especially among those who reported sleeping for fewer than six hours per day. This difference, although subtle, suggests a possible relationship between sleep disturbances and nutritional status, which is consistent with previous studies linking sleep restrictions to metabolic imbalances and weight gain (Covassin et al., 2022; van de Langenberg et al., 2019).

In emotional and psychological terms, one of the most consistent findings was the significant difference in the levels of depersonalisation between the groups compared. Those who slept for fewer than six hours had higher levels of this dimension of burnout, suggesting greater emotional exhaustion, greater emotional distance from patients, and a more cynical or defensive attitude towards their work. This result is concerning, considering the potential impact of depersonalisation on the quality of care and patient safety, as previous research has already pointed out (Khan et al., 2018; Sørengaard et al., 2021). In terms of fatigue, physical symptoms, and cognitive problems consistent with stress, the results obtained would be consistent with research reporting an increased fatigue, physiological alterations, memory loss, and difficulty responding to stimuli (Caruso, 2014; Gander et al., 2019; Urbón & Salavera, 2023). Along the same lines, studies such as those carried out by Yeo and Koh (2022) and Gifkins et al. (2020) have demonstrated the harmful effects of shift work, which is associated with an increased risk of cardiovascular problems, gastrointestinal disorders, and cognitive impairment—symptoms that were also evident in the data from this study.

Furthermore, although it may seem counterintuitive, the participants who reported sleeping for more than six hours had higher levels of ineffectiveness and interpersonal distrust. This could be because more sleep does not necessarily reflect restful sleep, but in some cases could be a symptom of avoidance, chronic fatigue, or underlying emotional distress. In this sense, feelings of ineffectiveness—expressed as low self-esteem, insecurity, or lack of control over one’s life—may not be directly determined by the hours of sleep, but by contextual factors such as the workload, social support, or self-imposed expectations. This phenomenon has been observed in studies linking excessive sleep to depressive symptoms and a lower quality of life (Sachdeva & Goldstein, 2020; Lee et al., 2024; Zhang et al., 2024). However, other studies suggest that the relationship between sleep duration and psychological well-being is complex and may vary depending on the individual, highlighting the importance of considering personal factors in sleep assessments (Sørengaard et al., 2021).

In relation to eating behaviours, the results show that participants who slept for fewer than six hours had a greater tendency towards dietary restrictions. This behaviour could be influenced by high levels of stress and anxiety, which alter emotional regulation and, consequently, eating habits. In turn, restrictive behaviour can intensify psychological distress, creating a vicious cycle of sleep deprivation, stress, and dysfunctional eating patterns. These findings are consistent with previous research linking insufficient sleep to metabolic disturbances and an increased risk of obesity. Studies such as those by Ko et al. (2007), Itani et al. (2017), Givens et al. (2015), and Liu and Chen (2020) indicate that sleeping for fewer hours is associated with a higher body mass index (BMI) and symptoms of eating disorders—data that were also observed in the present sample. Research such as that conducted by Spiegel et al. (2004), Taheri et al. (2004), and Franco et al. (2012) has explained this relationship based on hormonal appetite dysregulation caused by sleep deprivation, which supports the eating symptoms detected. Other studies have emphasised that sleep deprivation can lead to the increased consumption of high-calorie foods and increase the risk of developing eating disorders (Penev, 2007; Chaput & Tremblay, 2009; Moreno & Martorell, 2021). De Martino (2013) and Rovira-Llopis et al. (2024) provided a neurophysiological perspective, describing how alterations in the suprachiasmatic nucleus—the centre that regulates the circadian rhythm—can be affected by shift work, altering both sleep and metabolic patterns. Along the same lines, Arble et al. (2009) linked the timing of food intake with metabolic dysregulation, which is particularly relevant in night or shift work contexts such as that of this sample. In contrast, acute sleep deprivation does not necessarily alter ghrelin or leptin levels (Gresser et al., 2025), so further research is needed to clarify discrepancies. However, other studies, such as those by Jakubowicz (2013) and Caruso (2014), while not ruling out the effects of sleep, suggest that obesity and metabolic problems may be more related to meal times or work shift patterns than to the number of hours slept per se. This introduces a critical perspective that suggests interpreting the results with caution and considering multiple variables in the relationship between sleep and metabolic health.

Emotional and cognitive symptoms were significantly more frequent in the group of participants who slept for fewer than six hours per night, a finding that is consistent with previous research (Brand et al., 2021; Dong et al., 2022). Among the most commonly reported symptoms were persistent fatigue, memory loss, difficulty concentrating, negative thoughts about oneself, tachycardia, and dermatological disorders. This set of manifestations suggests that sleep deprivation has a broad and profound impact, simultaneously affecting physical, emotional, and mental functioning. In particular, the high incidence of depressive symptoms in this group highlights the importance of considering sleep as a fundamental pillar of psychological well-being. In addition, significant differences were found in stress-coping strategies: people with less night-time rest tended to resort more frequently to social isolation and self-medication. These strategies, which are generally maladaptive, may reflect failed attempts to manage accumulated distress, as recent studies have indicated (Brand et al., 2021; Dumont & Paquet, 2022). However, it has also been pointed out that excessive sleep could be equally problematic. Some research has suggested that sleeping more than necessary could predict long-term cognitive decline (Yu et al., 2024; Basta et al., 2024). This duality in the effects of sleep—both by defect and by excess—highlights the need for further research into its role in mental and cognitive health from a comprehensive perspective.

From an explanatory perspective, the regression analyses conducted in this study showed that a set of personal, work-related, and behavioural variables contribute significantly to explaining differences in the number of hours of sleep. This finding is consistent with previous research that has pointed to the role of sleep deprivation in the onset of emotional regulation difficulties and depressive symptoms (Brand et al., 2021; Dong et al., 2022). In particular, this includes the presence of allergies (possibly as somatic manifestations of stress), marital status (recent studies confirm that in the case of men, having a partner reduces their stress levels, whereas in the case of women, the opposite is true; Fivecoat et al., 2024), the number of hours worked per week, the levels of depersonalisation, alcohol consumption, interpersonal distrust, and skipping meals. Together, these variables explained 18% of the variance in sleep duration. Although this percentage does not provide a complete explanation of the phenomenon, it does identify a clinically significant pattern, suggesting the existence of a network of psychosocial factors that directly affect night-time rest. These results reinforce the need to approach sleep from a multidimensional perspective that considers not only individual aspects, but also behavioural, cultural, and contextual factors (e.g., family responsibilities).

A particularly relevant finding of the present study was the relationship between allergies and sleep, as this symptom—often overlooked in the study of eating disorders—was identified as the most powerful predictor within the model. This result represents a novel contribution to the field of eating disorders and comprehensive health. The connection between allergies and sleep could be explained by the impact of chronic stress on the immune system, which, when altered, can manifest itself in somatic symptoms such as allergic reactions. These, in turn, could interfere with the quality and quantity of sleep, hindering restful sleep (Moore & Collins, 2022). At the same time, depersonalisation and alcohol consumption emerged as significant variables. These factors suggest that emotional distress and attempts at self-regulation through substance use may play an important role in reducing the duration and quality of rest. However, not all studies agree with this interpretation. Some authors have cautioned that the relationship between stress, the immune system, and sleep remains complex and not entirely conclusive, pointing to the need for further research on these links from an integrative perspective (Prather et al., 2015; Bae et al., 2019; Garbarino et al., 2021).

Finally, a network analysis allowed for a more integrated representation of how the most relevant factors related to rest are interconnected. In the group of workers who slept for fewer than six hours, a particularly dense and complex network was identified, characterised by multiple positive correlations between variables such as alcohol consumption, the presence of allergies, depersonalisation, skipping meals, and interpersonal distrust (Al Harbi et al., 2023; Creasy et al., 2022; Shechter et al., 2023). This pattern suggests that emotional, physical, and behavioural distress tend to operate in an interconnected manner, with factors that not only coexist, but also appear to reinforce each other, creating a broader state of vulnerability. In contrast, among professionals who slept for longer, the connections between variables were significantly weaker or even negative, which could reflect a more balanced personal and work environment with less risk of emotional dysregulation. However, it is important to note that these associations, while revealing, do not necessarily imply causal relationships. As several authors have pointed out, this type of analysis may be influenced by external variables that are not considered or controlled, which requires caution in interpreting the results (Prather, 2019; Patel & Hu, 2020).

In summary, the results of this study highlight the importance of sleep as a key indicator of well-being among healthcare professionals. Sleeping for fewer than six hours is consistently associated with higher levels of emotional, physical, and behavioural vulnerability.

### 4.1. Practical Implications

The results obtained allow us to identify a set of personal, work-related, and psycho-emotional variables that significantly affect the amount of sleep that healthcare workers get, which in turn is related to multiple indicators of psychological distress and unhealthy behaviours. From an applied perspective, these findings have important practical implications, both for the design of interventions aimed at promoting the well-being of healthcare personnel and for the formulation of organisational policies that favour healthy work environments.

Firstly, the data show that sleeping for fewer than six hours is associated with higher levels of depersonalisation, physical and emotional symptoms of stress, a higher prevalence of dysfunctional self-care behaviours (such as self-medication or social isolation), and worse indicators on scales related to mental health, such as the EDI diet index and the EAT-40 subscales of interpersonal distrust and inefficacy. These results underscore the need to consider sleep not only as a basic physiological aspect, but also as a key indicator of emotional balance, perceived self-efficacy, and the ability to form social bonds in demanding work contexts.

From an organisational point of view, healthcare institutions should develop strategies to prevent chronic sleep deprivation, especially in rotating or night shifts, through measures such as planned shift rotations, adjusting the number of hours worked per week and reviewing the workload. Similarly, the results of the linear regression suggest that the number of hours worked per week and marital status are relevant predictors of night-time rest, which may guide differentiated interventions according to the sociodemographic profile or the contractual status of staff.

Furthermore, the finding that alcohol consumption, skipping meals, and the presence of allergies (as a physiological symptom of stress) are significantly associated with sleep restrictions highlights the need for comprehensive occupational health programmes that incorporate not only clinical or psychological interventions, but also nutritional education, the early detection of addictions, and physical stress management. The relationship observed between depersonalisation and interpersonal distrust, mediated by hours of sleep, reveals that fatigue and a lack of rest could erode the quality of treatment between colleagues, and even towards patients, thus affecting the working environment and the quality of care.

A visual representation through a network analysis reinforces this need for a systemic and interdisciplinary approach. Positive correlations between variables such as alcohol consumption, depersonalisation, allergies, and skipping meals in workers who sleep for fewer than six hours indicate a pattern of cumulative psychosocial vulnerability, which could escalate to clinical pictures of burnout, anxiety, or affective disorders if not detected and addressed in a timely manner.

In this sense, the results also allow risk groups to be established within healthcare personnel, such as workers with more than five years’ seniority, women with children, or single people with high levels of emotional symptoms and a low self-efficacy. For these groups, the design of selective intervention strategies would be particularly relevant, such as sleep hygiene workshops, emotional support spaces, occupational psychology services, or changes in shift allocation.

At the macro level, the implications of this study can serve as a basis for changes in healthcare labour legislation, aimed at guaranteeing a minimum number of hours of restful sleep, recognising rest as a worker’s right and not just a matter of individual health. As the data show, insufficient rest has both physical and psychological repercussions, affecting the productivity, motivation, and problem-solving ability of healthcare personnel, which could have direct consequences for patient safety. Sleep should be considered a key variable in the assessment of psychosocial risk in hospitals, not only because of its impact on the well-being of professionals, but also because of its effect on the quality of care provided to patients.

### 4.2. Limitations of the Study

It is important to recognise a number of methodological and contextual limitations that limit the scope and generalisation of the findings. Firstly, this study was based on a cross-sectional design, which prevents causal relationships between variables from being established. Although significant associations were identified between various predictor variables and hours of sleep, it cannot be stated with certainty whether it was lack of sleep that caused the psychological and physical alterations observed, or whether these same alterations influenced the quality of rest. Future longitudinal studies could help clarify these temporal and causal relationships.

Secondly, data collection was carried out using self-reports, which implies a possible social desirability bias or underestimation of behaviours that could be considered negative, such as self-medication, alcohol consumption, or diet in the EAT-40 questionnaire. Although the anonymity of the responses was guaranteed, it cannot be ruled out that some participants may have responded more favourably or neutrally than is actually the case.

Thirdly, from a methodological perspective, the lack of control of extraneous variables—such as sleep disorders or psychiatric conditions—may distort the relationship between independent and dependent variables, generating spurious associations or biases in the internal validity of the study.

Another important limitation lies in the representativeness of the sample. Although the total number of participants was adequate for the analyses performed, the sample was overrepresented by women and nursing staff, which may have limited the extrapolation of the results to other professional categories or populations with greater gender diversity. Unfortunately, this is still a profession with a high representation of women. In future research, it would be advisable to have a more balanced sample in terms of gender and with the greater participation of doctors and other professional profiles in the healthcare field, as well as to conduct further research on the influence of social and work expectations on the gender emotional balance or whether family pressures determine part of emotional well-being.

Likewise, the category of hours of sleep was divided dichotomously (<6 h vs. ≥6 h); although this simplified the analysis, it may have limited the sensitivity for capturing intermediate differences. Subsequent studies could benefit from the use of more detailed and continuous measures of sleep, which would provide more accurate information on sleep patterns.

Finally, although numerous psychosocial, occupational, and clinical variables were considered, other potentially relevant dimensions, such as the perceived social support, job satisfaction, coping style, or sleep quality, were not included in the model. Their incorporation in future studies could enrich the understanding of the mechanisms linking sleep hours to the psychological well-being of healthcare personnel.

## 5. Conclusions

For the first time, allergies, which are considered somatic manifestations of stress, were identified as the most powerful predictor of insufficient sleep in healthcare workers. This association had not been previously documented and represents a significant advance in the field. The results show how somatic (allergies), emotional (depersonalisation), and behavioural (skipping meals, alcohol, interpersonal distrust) factors interact as a web of cumulative psychosocial vulnerability, beyond simple statistical associations. It was also observed that sleeping for more than six hours does not guarantee recovery; on the contrary, it was linked to a greater perception of ineffectiveness and interpersonal distrust, suggesting that excessive sleep could reflect chronic fatigue or underlying emotional distress.

Overall, insufficient sleep emerges as a critical marker of the emotional and functional well-being of healthcare professionals. This finding opens up a new field of research focused on the interrelationship between chronic stress, the immune system, and sleep, as previous studies have shown how allergic rhinitis negatively impacts sleep quality and increases the symptoms of fatigue or depression, and how a lack of sleep is associated with a greater sensitivity to allergens.

It is imperative that further research explores variables linked to the actual sleep quality (not just duration), communication styles in the workplace, job demands, the psychosocial environment, and professional personality. These variables could enhance the protection of healthcare workers’ mental health more efficiently. It is recommended that those responsible for designing occupational health policies implement strategies to promote restorative rest, including sleep hygiene and allergen-free environments (cleanliness and adequate ventilation), emotional and nutritional education programmes to prevent dysfunctional behaviours (self-medication, isolation, skipping meals), and the consideration of sleep as an indicator of psychosocial health and quality of care, not just as a biological need.

## Figures and Tables

**Table 3:** Stress symptoms according to hours of sleep.

	*Fewer Than Six Hours* *(n = 163)*	*More Than Six Hours* *(n = 31)*	*OR* *(95% CI)*	*p*
Memory loss	77 (39.7) *	14 (7.2)	1.08 (0.5–2.35)	0.04
Negative self-talk	45 (23.2) *	8 (4.1)	1.09 (0.45–2.62)	0.04
Difficulty concentrating	68 (35.1) ^(^*^)(^**^)^	13 (6.7)	0.99 (0.45–2.15)	0.001
Fatigue	82 (42.3) *	15 (7.7)	1.08 (0.5–2.32)	0.03
Tachycardias or arrhythmias	30 (15.5) ^(^*^)(^*^)^	6 (3.1)	0.94 (0.35–2.49)	0.016
Skin problems	44 (22.7) ^(^*^)(^*^)^	8 (4.1)	1.06 (0.44–2.55)	0.019
Isolation	18 (9.3) *	3 (1.5)	1.15 (0.32–4.19)	0.05
Crying easily	29 (14.9) *	6 (3.1)	0.9 (0.33–2.39)	0.04
Easily discouraged	40 (20.6) *	7 (3.6)	1.11 (0.44–2.78)	0.04
Self-medication	15 (7.7) ^(^*^)(^*^)^	3 (1.5)	0.94 (0.25–3.48)	0.007

Note: Cells represent absolute frequencies and percentages of the total. OR: Odds ratio; 95% confidence interval = * *p* < 0.05; ** *p* < 0.01.

**Table 4:** Linear regression test in relation to hours of sleep.

	*B*	*e.t.*	*R* ^2^	*t*	*Sig*	*Durbin–Watson*
Constant	1.531	0.113		13.586	<0.001	1.896
Allergies	−0.175	0.067	0.039	−2.604	0.010	
Marital status	0.082	0.033	0.072	2.505	0.013	
Hours worked per week	0.007	0.002	0.094	2.749	0.007	
Depersonalisation	−0.019	0.006	0.117	−2.937	0.004	
Weekly alcohol consumption	0.099	0.042	0.139	2.340	0.020	
Interpersonal mistrust	0.028	0.012	0.161	2.346	0.020	
Food jumps	−0.109	0.053	0.180	−2.041	0.043	

## Data Availability

The data presented in this study are available from the corresponding author upon request.
